# Persistent postural perceptual dizziness is on a spectrum in the general population

**DOI:** 10.1212/WNL.0000000000009373

**Published:** 2020-05-05

**Authors:** Georgina Powell, Hannah Derry-Sumner, Deepak Rajenderkumar, Simon K. Rushton, Petroc Sumner

**Affiliations:** From the School of Psychology (G.P., S.K.R., P.S.), Cardiff University; and University Hospital of Wales (H.D.-S., D.R.), Cardiff, UK.

## Abstract

**Objective:**

To examine the idea that symptoms of persistent postural perceptual dizziness (PPPD) are more common than previously assumed and lie on a spectrum in the general population, thus challenging current theories that PPPD is only a consequence of a vestibular insult.

**Methods:**

We collected 2 common clinical questionnaires of PPPD (Visual Vertigo Analogue Scale [VVAS] and Situational Characteristics Questionnaire [SCQ]) in 4 cohorts: community research volunteers (n = 1941 for VVAS, n = 1,474 for SCQ); paid online participants (n = 190 for VVAS, n = 125 for SCQ); students (n = 204, VVAS only); and patients diagnosed with PPPD (n = 25).

**Results:**

We found that around 9%, 4%, and 11%, respectively, of the 3 nonclinical cohorts scored above the 25th percentile patient score on 1 PPPD measure (VVAS) and 49% and 54% scored above the 25th percentile patient score on the other measure (SCQ). Scores correlated negatively with age (counter to expectation). As expected, scores correlated with migraine in 2 populations, but this only explained a small part of the variance, suggesting that migraine is not the major factor underlying the spectrum of PPPD symptoms in the general population.

**Conclusion:**

We found high levels of PPPD symptoms in nonclinical populations, suggesting that PPPD is a spectrum that preexists in the population, rather than only being a consequence of vestibular insult. Atypical visuo-vestibular processing predisposes some individuals to visually induced dizziness, which is then exacerbated should vestibular insult (or more generalized insult) occur.

Persistent postural perceptual dizziness (PPPD) is a chronic functional condition that describes dizziness and nonspinning vertigo induced by self-movement, challenging visual environments, and upright posture.^1,2^ PPPD was recently defined in order to unite a range of related conditions previously known as supermarket syndrome,^3^ visual vertigo,^4^ chronic subjective dizziness,^5^ space and motion discomfort,^6^ and phobic postural vertigo.^7^ Common triggers include situations of vestibulo-visual conflict, such as cinemas, and intense visual environments, such as supermarkets. Patients often develop functional gait abnormalities and an excessive vigilance about balance sensations.^8–10^ PPPD is the second most common condition reported in dizziness clinics and is particularly prevalent in middle age.^11,12^

When PPPD presents in clinic, the history often involves an acute vestibular insult, such as labyrinthitis or benign paroxysmal positional vertigo.^1,4,13^ One hypothesis is that the brain adapts to the vestibular insult by becoming more reliant on visual information about self-movement. This visual dependency remains and leads to dizziness when visual motion cues are particularly salient.^4,10,14–16^ Another related hypothesis suggests that PPPD is caused by a failure of the postural control system to adapt, which leads to problems predicting the sensory consequences of self-movement.^7,17^ However, it remains a puzzle why some patients develop PPPD and other patients do not, despite similar vestibular insults. Here we explore the possibility that subclinical PPPD symptoms may exist on a spectrum in the population regardless of vestibular insult. If so, these preexisting symptoms could predispose an individual to exacerbated and debilitating PPPD if a vestibular insult does occur.

The general prevalence of PPPD is difficult to estimate. A study of individuals registered with general practitioners in the United Kingdom found that around 4% of the population experience some form of chronic dizziness, although not all of these will be related to PPPD.^18^ In our own experience, self-reported symptoms of PPPD appear even more common than 4%. Once one starts discussing the topic, it is remarkable how many people report feeling dizzy in the types of situations that are associated with PPPD, despite never seeking medical advice. These observations led us to hypothesize that subclinical symptoms of PPPD, or even undiagnosed cases, are much more prevalent in the general population than previously acknowledged.

Prior evidence is lacking for whether PPPD is generally caused by or generally predates, vestibular insults, or both. Recent neuroimaging studies report that patients with PPPD show differences in brain structure, connectivity, and function in regions related to visual, vestibular, and spatial processing.^19–23^ However, such studies are unable to disentangle preexisting differences from adaptive changes following acute vestibular insults.

To test whether PPPD symptoms exist as a spectrum in the general population, over 2,000 people completed the Visual Vertigo Analogue Scale (VVAS)^24^ and the Situational Characteristics Questionnaire (SCQ),^25^ 2 questionnaires designed to categorize patient symptoms in clinics. We then compared the responses from the general population with those of patients diagnosed with PPPD. We also collected information on co-occurrence of migraine (a known associate of PPPD) and other common vestibular conditions in our participants. Since the 2 PPPD questionnaires were developed in clinical populations, we also report their internal consistency in a general population sample.

## Method

### Participants

Our participant sample comprised 4 cohorts:

1. General population: An advertisement to complete the survey was sent via email to 18,683 members of a community public health participant list in Wales. The survey was advertised as being about “Health and the Senses” and contained the following text:

The School of Psychology at Cardiff University are investigating health and the senses through an online survey. Dizziness is common in the general population and can have serious consequences for daily functioning and health. The research team are interested in a particular type of dizziness that is triggered by being in certain environments. These tend to be environments where there is a lot of clutter, e.g., a supermarket or a crowded street. They are interested in how common this dizziness is in the general population and how it might relate to other conditions (e.g., migraines). In the future, they hope this research will help them to develop more effective rehabilitation tools for dizziness. The online survey will include questions and pictures about sensory sensitivity, dizziness and migraines, and is open to everyone. They would like to hear from a range of people, whether or not you suffer from dizziness and migraines.

We emphasized the inclusivity of the survey so that individuals with an interest in dizziness and migraines would not selectively participate, although there is inevitably some bias in any sample recruited through an advertisement. Out of the total participant list, 2,280 respondents filled out some or all of the survey. We received complete data on the VVAS from 1,941 respondents and complete data on the SCQ from 1,474 respondents. The average age of participants was 55 years (SD, 15.2; range, 17–88) and 74% were female. Average level of education attainment was 2.9 (SD, 1.3; where 0 = no education, 1 = GCSE/O-level equivalent, 2 = A-level/BTEC equivalent, 3 = undergraduate, 4 = postgraduate). No payment or compensation was offered to these participants.

1. Paid participants: 211 participants were recruited from Prolific Academic, a website where members of the general public can sign up to take part in studies for payment. The advertised study title was “How sensitive are your senses?” All respondents provided responses on at least one of the questionnaires, 190 on the VVAS and 125 on the SCQ. The average age of participants was 27 years (SD, 7.5; range 18–68) and 30% were female. Average level of education attainment was 2.6 (SD, 0.9). Participants were paid £5.75 each.
2. Psychology students: 204 undergraduate students at Cardiff University completed the VVAS only as part of a battery of questionnaires completed by nearly all students in the cohort at the beginning of their course. Thus there was no self-selection in response to an advertisement. The average age of participants was 19 years (SD, 1.6; range 18–30) and 85% were female.
3. Patients with PPPD: 25 patients were recruited from the vestibular clinic at University Hospital Wales (UHW). All patients had received a diagnosis of PPPD from a clinical scientist in audiology or a consultant audiovestibular physician, following common tests to examine vestibular functioning, including Halmagyi bedside head thrust testing, video head impulse testing using the Synapsys [Marseille, France] system, videonystagmography (typically saccades, pursuit, and gaze using the ICS Chartr 200 system [natus, Pleasanton, CA]), and caloric testing if necessary. Some patients had additional vestibular conditions (table 1). The average age of participants was 44 years (SD, 14.3; range 11–67) and 60% were female. Average level of education attainment was 3 (SD, 1.4). We had complete data from all 25 patients on the VVAS and from 20 patients on the SCQ. Patients received no payment or compensation.

**Table 1:**
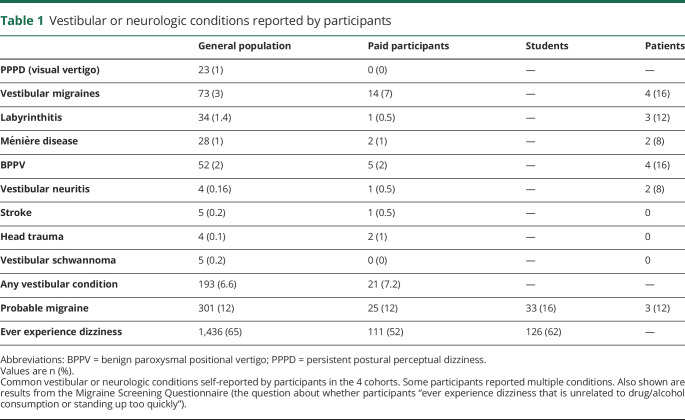
Vestibular or neurologic conditions reported by participants

### Standard protocol approvals, registrations, and patient consents

Ethical approval for the 3 general population samples was granted by the School of Psychology, Cardiff University ethics committee. Ethical approval for the patient population was granted by Cardiff and the Vale University Health Board. All participants completed an electronic consent form.

### Materials

All aspects of the survey were delivered via Qualtrics, an online survey tool.

#### Demographic information

We collected basic demographic information about age, sex, and educational attainment. We also asked participants if they ever “experienced dizziness that was unrelated to alcohol or drug consumption or standing up too quickly” (e-Results, osf.io/gpjd5/?view_only=c110b21c59254cc6aae260c236ac534f). Participants in the general population and paid cohort were asked to report if they had a current diagnosis of any common vestibular conditions (data shown in table 1) and to rate if they experienced motion sickness while travelling in moving vehicles on a scale of 1–5 (e-Results, osf.io/gpjd5/?view_only=c110b21c59254cc6aae260c236ac534f).

#### Visual Vertigo Analogue Scale

The VVAS is a 9-item questionnaire that asks respondents to rate on a scale from 0–10 the amount of dizziness they experience in situations that are known triggers for patients with PPPD.^24^ These include walking down a supermarket aisle, walking across a patterned floor, and going to the cinema. The item scores are then averaged, and this score is multiplied by 10. The maximum score is 100. It has previously been validated on a group of patients with vestibulopathy^24^ with a comparison group of people receiving outpatient orthopedic physiotherapy, but is currently untested in a general population sample.

#### Situational Characteristics Questionnaire

The SCQ was originally developed as a measure of space and motion discomfort, which is now considered to be closely related to the new diagnosis of PPPD.^1,25^ The SCQ is a 20-item questionnaire that, like the VVAS, also asks about discomfort in situations of intense visual salience of visual-vestibulo conflict. Situations are rated between 0 and 3 and scores are normalized by subtracting responses to paired situations that are not commonly associated with visually induced dizziness. The final score is obtained by dividing the summed ratings across all items by the total number of items and multiplying by 10; therefore, the maximum score is 30. Item 15 from the paid participant survey responses was removed due to a question transcription error.

#### Migraine Screening Questionnaire

The Migraine Screening Questionnaire is a 5-item screening tool that identifies probable migraine. Participants answer yes/no questions about headache episodes they experience, which include “Do you usually suffer from nausea when you have a headache?” and “Does light or noise bother you when you have a headache?”^26^ Participants must respond “yes” to 4 or more of the 5 questions to obtain a result of probable migraine.

#### Hospital Anxiety and Depression Scale

The Hospital Anxiety and Depression Scale (HADS) is a 14-item scale containing 7 questions that contribute to an anxiety subscale and 7 questions related to a depression subscale.^27^ Due to the previous focus on anxiety and dizziness, we only used the anxiety subscale. Example anxiety questions include “I feel tense or wound up” and “I get a sort of frightened feeling as if something awful is about to happen.” Participants are given 4 response options per question (e.g., most of the time, a lot of the time, from time to time, not at all) and are asked to select the option that is closest to how they have been feeling in the past week. Questions are both positively and negatively worded. Options are scored from 0 to 4, where 4 indicates more anxiety. Items can then be summed to provide an anxiety subscale score and depression subscale score.

### Procedure

General population participants were emailed an advertisement and link to the survey and were free to complete it in their own time and on their own devices. Paid participants were free to sign up to the study on Prolific Academic and then received a link to the survey to complete on their own devices. Student participants completed the questionnaire in a large computer laboratory during one of their courses. All participants were given an information page to read before the start of the survey and then asked to sign an electronic consent form. A debrief page was shown at the end of the survey that contained more details about the study.

Participants from the patient cohort attended an appointment at the vestibular clinic at UHW following a referral from their general practitioner, an audiologic physician, or an ear, nose, and throat surgeon. During the visit, they discussed their dizziness symptoms with a clinical scientist and completed vestibular tests. Once the diagnosis of PPPD had been confirmed by the clinical scientist at the end of the appointment, they were given the option to consent and a link to complete the survey online at their own convenience.

### Data availability

All data reported in this article will be available on the Open Science Framework following acceptance.

## Results

### Internal consistency of VVAS and SCQ

The VVAS showed excellent internal consistency with a Cronbach α of 0.91 in the general population cohort, 0.86 in the paid participant cohort, 0.87 in the student population cohort, and 0.86 in the patient cohort. The SCQ had acceptable internal consistency with an α of 0.79 in the general population, 0.65 in the paid participant sample, and 0.78 in the patient cohort (the student cohort did not complete the SCQ).

### Population distributions for VVAS and SCQ

**Figure 1:**
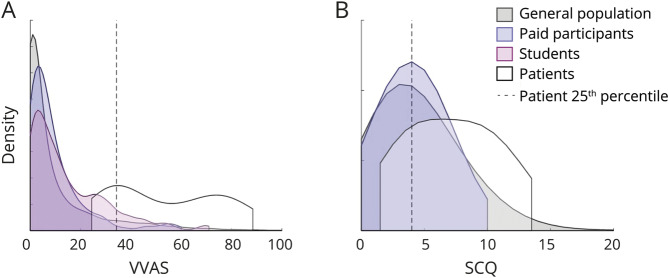
Spectrum of persistent postural perceptual dizziness (PPPD) symptoms in patients and nonclinical participants Kernel density plots show spectrum of PPPD symptoms (A = Visual Vertigo Analogue Scale [VVAS], B = Situational Characteristics Questionnaire [SCQ]) in the 4 participant cohorts: general population, paid participants, students, and patients.

Participants in the 3 nonclinical samples with any self-reported vestibular conditions were removed from the analyses (general population, n = 193; paid participants n = 21; see supplementary data for overlap with patients, osf.io/gpjd5/?view_only=c110b21c59254cc6aae260c236ac534f). Participants with probable migraine were not excluded, but those with reported vestibular migraine were excluded. Kernel density plots of population distributions for the 4 participant cohorts are shown in figure 1. Although the modal score on the VVAS for all nonclinical participants is 0, there is a smooth spread of scores across much of the scale. Furthermore, 9% of participants in the general population cohort, 4% in the paid participant cohort, and 11% in the student cohort scored at or above the 25th percentile patient score (dashed line); 13%, 9%, and 23%, respectively, scored above the minimum patient VVAS score. For the SCQ, 49% of participants in the general population cohort and 54% in the paid participant cohort scored at or above the 25th percentile patient score, and 83% and 86%, respectively, scored above the minimum patient score.

### Correlation between the 2 PPPD symptoms measures: VVAS and SCQ

**Figure 2:**
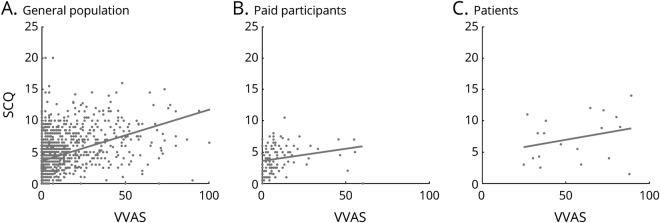
Relationship between Visual Vertigo Analogue Scale (VVAS) and Situational Characteristics Questionnaire (SCQ) scores Scatterplots show a significant positive relationship between VVAS and SCQ scores in the general population (A), paid participants (B), and patients (C).

There was a positive correlation between the VVAS and SCQ in the general population cohort (*R*_*S*_[1,427] = 0.52 [*p* < 0.001]) and paid participant cohort (*R*_*S*_[122] = 0.41 [*p* < 0.001]; figure 2). It is worth noting that although these correlation coefficients are moderate to large, it is not the case that the 2 measures are close to collinear. The correlation between the VVAS and SCQ in the patient cohort was not significant (*R*_*S*_[18] = 0.27). The relationship trended in the same direction so this could be attributed to a relatively small sample size.

### Association among VVAS, SCQ, and migraine

Migraine is known to be associated with PPPD, and we were interested in whether the VVAS and SCQ identified this relationship in a nonclinical sample. Logistic regressions were conducted to explore whether the VVAS and SCQ significantly predicted the incidence of migraine. For the general population cohort, the logistic regression model was statistically significant (χ^2^[2] = 101 [*p* < 0.001]). The model explained 10% (Nagelkerke *R*^*2*^) of the variance in migraine and correctly predicted 69% of migraine cases (area under the curve [AUC], 0.77). Both the VVAS and SCQ significantly predicted the incidence of migraine (*p* < 0.001). See table 2 for a full breakdown of model coefficients.

**Table 2:**
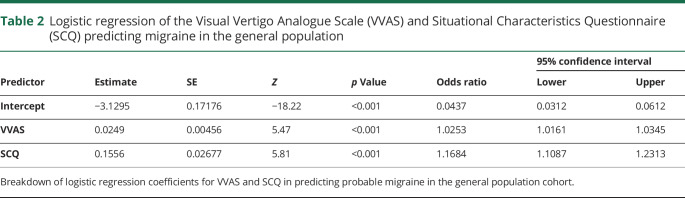
Logistic regression of the Visual Vertigo Analogue Scale (VVAS) and Situational Characteristics Questionnaire (SCQ) predicting migraine in the general population

For the paid participant cohort, the logistic regression model was not significant (χ^2^[2] = 2.6). Neither the VVAS or SCQ significantly predicted the incidence of migraine (table 3). For the student population, only data from the VVAS were available. Overall the model was significant (χ^2^[1] = 12 [*p* < 0.001]), and explained 6.8% (Nagelkerke *R*^*2*^) of the variance in migraine and correctly predicted 63% of migraine cases (AUC, 0.69). See table 4 for full breakdown of model coefficients.

**Table 3:**
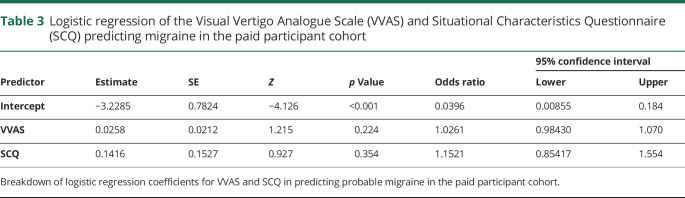
Logistic regression of the Visual Vertigo Analogue Scale (VVAS) and Situational Characteristics Questionnaire (SCQ) predicting migraine in the paid participant cohort

**Table 4:**
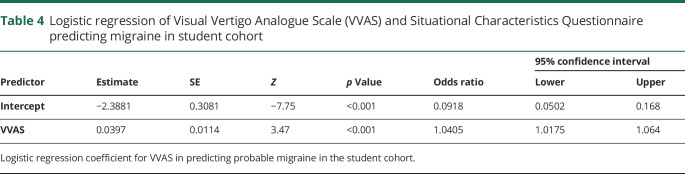
Logistic regression of Visual Vertigo Analogue Scale (VVAS) and Situational Characteristics Questionnaire predicting migraine in student cohort

Taken together, these findings suggest that individuals with more symptoms of PPPD are also more likely to report experiencing migraines, and this is the case even when they do not report the presence of vestibular migraines (as these participants were excluded from our sample). This is consistent with previous findings of an overlap between patients with PPPD and migraine,^1,5,13^ and suggests that this relationship holds in a nonclinical sample.

**Figure 3:**
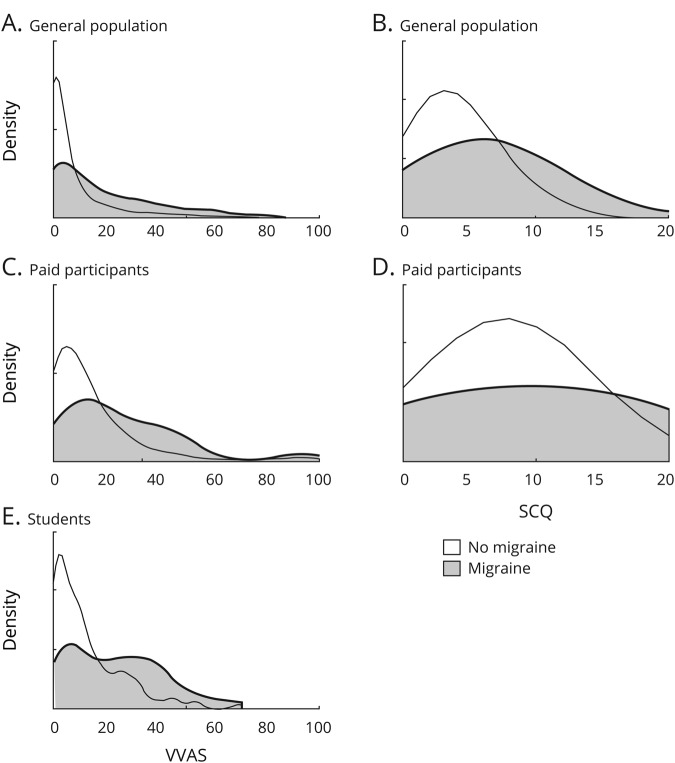
Comparison between participants with and without migraine on the Visual Vertigo Analogue Scale (VVAS) and Situational Characteristics Questionnaire (SCQ) Density plots show distributions of migraine (gray) and no migraine (white) cases for the general population cohort (A, B), paid population cohort (C, D), and student cohort (E) for the VVAS (A, C, E) and SCQ (B, D). The student cohort did not complete the SCQ. Individuals reporting migraine are more likely to score highly on the VVAS and SCQ. However, the spreads of VVAS and SCQ scores are not explained entirely by those with migraine.

The presence of migraine does not entirely explain the spectrum of PPPD symptoms. Figure 3 shows the overlap in VVAS and SCQ scores for individuals with and without migraine, and although individuals with migraine tend to score higher on both scales, there is still a large variation in PPPD symptoms even in individuals without migraine. Therefore, it is clear that migraine is not the only factor associated with the spectrum of PPPD symptoms in the general population.

### Association among VVAS, SCQ, and anxiety and depression

Anxiety, and to some extent depression, can be heightened in patients with PPPD. We tested for correlation of anxiety and depression with PPPD symptoms in the subset of the general population and paid cohorts that provided complete responses to the HADS. The correlation of VVAS and anxiety was *R*_*s*_ (948) = 0.48 (*p* < 0.001) in the general population and *R*_*s*_ (123) = 0.43 (*p* < 0.001) in the paid cohort. The correlation of SCQ and anxiety was *R*_*s*_ (887) = 0.45 (*p* < 0.001) in the general population and *R*_*s*_ (123) = 0.32 (*p* < 0.001) in the paid cohort. The correlations of VVAS with depression in the general population and paid cohort were *R*_*s*_ (948) = 0.37 (*p* < 0.001) and *R*_*s*_ (123) = 0.29 (*p* < 0.001), respectively. The correlations of SCQ with depression in the general population and paid cohort were *R*_*s*_ (887) = 0.37 (*p* < 0.001) and *R*_*s*_ (123) = 0.21 (*p* < 0.05), respectively.

### Association among VVAS, SCQ, and basic demographics

The large general population sample had sufficient numbers and variance in age and other demographics to assess their correlations with VVAS or SCQ. We found negative correlations between age and both VVAS and SCQ (*R*_*s*_ [1,926] = −0.3 [*p* < 0.01] and *R*_*s*_ [1,463] = −0.23 [*p* < 0.01], respectively), which is surprising because previous studies have found that PPPD-related dizziness is common in middle age.^11^ If nonclinical PPPD symptoms are reported more in younger than older people, this might suggest that a vestibular insult preexists, given that the cumulative probability of having experienced a vestibular insult would increase with age. These results were not due to limited variation in age range in this cohort as the range spanned 17–88 years with a mean age of 55.

VVAS scores were significantly higher in female than male participants (*U* = 260,933, N_male_ = 500, N_female_ = 1,435 [*p* < 0.001, *n*^*2*^ = 0.04]) and so were SCQ scores (*U* = 115,465, N_male_ = 367, N_female_ = 1,102 [*p* < 0.001, *n*^*2*^ = 0.1]). Education achievement was not significantly related to either VVAS (*R*_*s*_ [1,939] = −0.02) or SCQ scores (*R*_*s*_ [1,470] = −0.01). VVAS and SCQ were correlated with motion sickness, but neither patients or high scorers in the general population necessarily report motion sickness (see supplementary information, osf.io/gpjd5/?view_only=c110b21c59254cc6aae260c236ac534f).

## Discussion

PPPD is a common chronic dizziness condition presenting in neurology and neuro-otology clinics and is characterized by a pattern of dizziness provoked by visual stimulation, posture, and self-movement.^1,2^ In this article, we explored the prevalence of subclinical symptoms of PPPD in nonclinical samples. We found that symptoms of PPPD are remarkably prevalent in the general population, with around 9% scoring above the 25th percentile patient score on 1 PPPD measure (VVAS^24^) and around 50% scoring above the 25th percentile patient score on the other measure (SCQ^25^). Furthermore, the distribution of symptoms was not bimodal; rather there was a smooth continuum in symptoms between the least and most severe cases. This suggests that a PPPD diagnosis is not categorical, but there is a large natural variation in symptoms even in nonclinical cohorts. We suggest that atypical visuo-vestibular processing predisposes some individuals to visually induced dizziness, which is then exacerbated should vestibular insult (or more generalized insult) occur. It may be important for clinicians to assess this in patients presenting with dizziness even if not the primary complaint, since it may predict a subsequent emergence of debilitating PPPD. If PPPD symptom scores are found to be high, a program of adaptation may reduce likelihood of evolution to full PPPD, though this idea remains untested.

Although we found that PPPD symptoms were fairly prevalent in our general population sample, we cannot know from our current research what the underlying cause for them might be. We speculate that the cause may lie in how visual systems process information and interface with other senses, rather than being triggered primarily by a vestibular insult. However, one possibility is that individuals in our sample with increased symptoms of PPPD might have had a past vestibular insult or other form of historical vestibular deficit. We were not able to carry out any clinical vestibular testing on the participants in our general population cohorts, so we are unable to rule this out. However, given that symptoms on the VVAS and SCQ were more common in younger participants than older participants, this suggests that they are not solely caused by a past vestibular insult, the probability of which would increase with age.

A second hypothesis would be that PPPD symptoms are associated with naturally varying visual dependence, since visual dependence is reported to vary naturally in the general population.^28^ Therefore, we might expect that individuals with naturally high visual dependence would be more likely to experience visually induced dizziness, particularly in situations of visual–vestibular conflict. These individuals might also be predisposed to develop severe symptoms of PPPD following an acute vestibular insult.^10^ Future research could examine this theory by exploring whether self-reported symptoms of PPPD are related to performance on classic measures of visual dependence, such as the rod and frame task^29^ and postural sway in response to optic flow stimulation.^30^

However, we do not believe that a simple construct of visual dependence could be the entire explanation. First, it is known that dependence on visual cues tends to increase with age,^31,32^ but here we found a negative correlation of PPPD symptoms and age. Second, visual dependence itself may be multifaceted, since different measures of visual dependence sometimes fail to correlate.^15^ Third, being dependent on vision would not necessarily produce symptoms of overload and dizziness if the visual system is providing veridical and reliable information.

PPPD in patients has also been associated with migraine.^1,5,13^ We found that this relationship was also present in our general population sample, but is by no means a full explanation for the observed spectrum. More research is needed on why and how migraine and PPPD are related. A further exacerbating factor could be sedentary lifestyle. If being prone to dizziness (or migraine) discourages activity, this in turn provides fewer opportunities for natural rehabilitation for PPPD symptoms.

PPPD in patients is also associated with psychiatric conditions such as anxiety and depression.^7,33–35^ These lie on a spectrum in the general population, so it might be that they contribute to increased PPPD symptoms in nonclinical populations. We found positive correlations with self-reported anxiety and depression. It is also possible that individuals with anxiety are more likely to report and seek help for their dizziness than those without anxiety, thus pseudoinflating the measured relationship between the 2 in patients.

One limitation of our study is that the majority of participants voluntarily filled out the questionnaires in response to an advertised survey. This could have introduced a degree of self-selection bias, whereby participants who experience more dizziness in day-to-day life may have a greater interest in their health and senses and be more likely to complete the survey. However, symptoms of PPPD were actually higher in the student cohort, where there was no advertisement, and nearly all students in a cohort participated as part of a large battery of questionnaires in a scheduled session for course credit. Therefore, we do not believe self-selection is the main reason for our observed rates of PPPD symptoms in nonclinical samples.

The majority of participants in 2 of our samples were female, but the paid sample was 70% male. This may explain why the number of participants with VVAS scores in the patient range was lower in the paid sample compared to the other samples, given that within the large sample, female respondents were more likely to report symptoms. This is consistent with a higher number of patients being female both here and in previous research.^1^

Both of the questionnaire measures we used, the VVAS and SCQ, were developed before the recent diagnosis of PPPD was agreed on by the Committee for the Classification of Vestibular Disorders of the Bárány Society.^1^ The VVAS was developed as a measure of visual vertigo symptoms^24^ and the SCQ was developed as a measure of space and motion discomfort.^25^ Both of these contributed to the united diagnosis of PPPD, and share some, but not complete, overlap in semantic content. However, we found that the correlation between the 2 measures, while significant, was not very strong. This may be a product of their history, which is based on different literature. The SCQ also relies on a difference score between competing situations. Difference scores can be advantageous to minimize overresponding biases, but they also carry statistical drawbacks for correlational analysis.^36^ The VVAS is based on absolute scores on single situations, which may partly explain why it had better internal consistency. The lack of strong collinearity might also reflect the ambiguity surrounding PPPD, where it is still not known if the condition represents one construct or comprises a number of subgroups.^1^

PPPD is a clinical diagnosis based on history, pattern of symptoms, clinical signs, and available test results (in this way, it is no different from other vestibular diagnoses such as Ménière disease or vestibular migraine). There is no objective test to confirm or disconfirm diagnosis and there are no data on interclinician agreement. Therefore, the false-positive rate is not known in patients and cannot be estimated for a subset of the general population who score highly on the questionnaires. What we can say is that some individuals are self-reporting the same symptoms in the same range of severity as do patients.

Subclinical symptoms of PPPD are remarkably common in the general population when measured using 2 common patient questionnaires. These symptoms might be expected to have some significant detrimental effects on day-to-day living. We hypothesized that subclinical symptoms of PPPD may be related to natural variation in levels of visual dependence in the general population, and could predispose individuals to develop severe PPPD symptoms should they experience a vestibular insult or brain insult. More research is needed to investigate the causes underlying natural variation in PPPD symptoms and examine whether preventative therapies might be useful for more severely affected individuals.

## Glossary

AUC: area under the curve
HADS: Hospital Anxiety and Depression Scale
PPPD: persistent postural perceptual dizziness
SCQ: Situational Characteristics Questionnaire
UHW: University Hospital Wales
VVAS: Visual Vertigo Analogue Scale
